# The cost of radiological procedures at Universiti Kebangsaan Malaysia Medical Centre: applying activity based costing methodology

**DOI:** 10.1186/1471-2458-14-S1-O21

**Published:** 2014-01-29

**Authors:** Roszita Ibrahim, Amrizal Muhammad Nur, Nor Haty Hassan, Hishamudin Am, Syed Mohamed Aljunid

**Affiliations:** 1 International Casemix & Clinical Coding Centre, UKM, 56000 Cheras, Kuala Lumpur, Malaysia; 2 United Nations University International Institute for Global Health (UNU-IIGH), 56000 Cheras, Kuala Lumpur, Malaysia; 3 Department of Radiology, UKMMC, 56000 Cheras, Kuala Lumpur, Malaysia

## Background

Department of Radiology is one of the intermediate cost centres for supporting patient treatment in wards and clinics in Universiti Kebangsaan Malaysia Medical Centre (UKMMC). A total of 121,221 procedures were performed in 2011. Unfortunately, since UKMMC began its operations, the actual cost of each procedure in this particular department has never been studied. Hence, it is important to determine the actual cost of each procedure in preparation for future autonomous university governance, particularly budgeting.

## Materials and methods

A cross-sectional study was conducted from January to December 2012 in all units in Department of Radiology. Activity Based Costing (ABC) methodology was used to analyse the cost of each procedure. Three hundred procedures had been collected using a micro costing form. Seven cost components were collected for each procedure: (1) staff salary (2) consumables (3) equipment (4) reagents (5) administration (6) maintenance (7) utilities. The results were analysed using SPSS version 22.0.

## Results

The five highest costs of radiological procedures had been identified. The highest mean cost procedures were Endovascular Interventional Radiology (EIR) (RM2214±1636), followed by Medical Nuclear (RM1741± 908), Magnetic Resonance Imaging (MRI) (RM1323±232), Computed Tomography (CT Scan) (RM395±152) and Mammogram (RM322±4). In addition, the highest component cost for EIR procedure were consumables (RM2782, 77%), for Medical Nuclear and MRI procedures, cost of equipment was the highest component which was RM875 (50%) and RM797 (60%), respectively. For CT scan procedures, cost of reagents was the highest component (RM143, 36%), which was followed by cost of consumables for Mammogram (RM775, 73%).

## Conclusions

An accurate and precise cost per radiological procedure can be determined by using ABC method. The important component that contributes to each of the activities performed at each procedure can be further justified.

